# Heart Failure With Improved Ejection Fraction in ACHD With Biventricular Physiology and Systemic Left Ventricle

**DOI:** 10.1016/j.jacadv.2026.103042

**Published:** 2026-07-21

**Authors:** Ahmed E. Ali, Zeyad Kholeif, Marwan Ahmed, Patricia A. Pellikka, Alexander C. Egbe

**Affiliations:** Department of Cardiovascular Medicine, Mayo Clinic Rochester, Rochester, Minnesota, USA

**Keywords:** echocardiography, heart failure, medical therapy, survival

## Abstract

**Background:**

In patients with heart failure (HF) with reduced ejection fraction (HFrEF) from acquired heart disease, guideline-directed medical therapy (GDMT) for HF is associated with improved left ventricular (LV) ejection fraction (LVEF) (ie, HF with improved EF (HFimpEF), and which in turn is associated with improved survival. Similar data are lacking in patients with congenital heart disease (CHD).

**Objectives:**

The objective of the study was to describe the prevalence, correlates, and prognostic implications of HFimpEF in adults with CHD, biventricular physiology, and systemic LV.

**Methods:**

Retrospective study of adults with CHD, biventricular physiology, and systemic LV presenting with HFrEF (2003-2023). Echocardiogram was performed at baseline and 1-year follow-up encounters. GDMT use was assessed at baseline and 1-year follow-up encounters using GDMT score. GDMT uptitration (ΔGDMT) was calculated as the difference between GDMT scores. HFimpEF was defined as HF with baseline LVEF ≤40%, and a subsequent absolute LVEF increase ≥10%, leading to LVEF >40% at follow-up echocardiogram. HFrEF relapse was defined as decline in absolute LVEF >10% leading to LVEF ≤40% in patients with HFimpEF. Cox regression analysis was used to assess the relationship between HFimpEF, GDMT score, and outcomes (death and cardiovascular events).

**Results:**

Of the 327 patients (age 46 ± 16 years; LVEF 31% ± 6%), 63 (19%) had HFimpEF. GDMT use (higher baseline and ΔGDMT) was associated with great odds of HFimpEF. HFimpEF was associated with a 26% decrease in all-cause mortality, and 28% decrease in cardiovascular events on multivariable analysis compared to patients without improvement in LVEF. Of the 63 patients with HFimpEF, 27% had HFrEF relapse, and HFrEF relapse was associated with higher risk of cardiovascular events.

**Conclusions:**

These data support the use and optimization of GDMT in this subgroup of CHD patients, and the need for ongoing clinical and imaging surveillance.

In the acquired heart disease population, heart failure (HF) is broadly classified based on left ventricular (LV) ejection fraction (EF) (LVEF), as HF with reduced EF (HFrEF) and HF with preserved EF (HFpEF).^1^^,^^2^ This classification has important clinical implications as HF management and outcomes differ between patients with HFrEF vs HFpEF.^1^, ^2^, ^3^, ^4^, ^5^, ^6^ Among patients with HFrEF, the use of guideline-directed medical therapy (GDMT) is associated with improvement in LVEF, and patients with improved LVEF (otherwise known as HF with improved EF [HFimpEF]) have lower risk of HF hospitalization and mortality compared to patients with HFrEF and HFpEF.^1^, ^2^, ^3^, ^4^, ^5^, ^6^ Although patients with HFimpEF have improved outcomes compared to patients with persistent HFrEF, the risk of mortality and HF-related morbidity in this group is significantly higher than that of patients without HF.^1^, ^2^, ^3^, ^4^, ^5^, ^6^ This suggests that improvement in LVEF with GDMT represents HFrEF remission rather than cure, and that HFimpEF may represent a distinct HF phenotype with a risk profile and natural history different from HFrEF and HFpEF.^1^, ^2^, ^3^, ^4^, ^5^, ^6^, ^7^

HF is the leading cause of mortality in adults with congenital heart disease (CHD).^8^^,^^9^ There are limited data about outcomes of GDMT in adults with CHD presenting with HF, but recent studies suggest that GDMT is associated with lower risk of HF hospitalization and mortality in this population.^10^, ^11^, ^12^, ^13^, ^14^, ^15^, ^16^, ^17^ A major limitation of prior studies is the inclusion of all CHD patients with HF regardless of LVEF, making it challenging to determine the effect of GDMT on LVEF, and whether patients with improved LVEF had lower risk of HF hospitalization and mortality.^10^, ^11^, ^12^, ^13^, ^14^, ^15^, ^16^, ^17^ The current study aims to address this knowledge gap, by describing the prevalence, correlates, and prognostic implications of HFimpEF in adults with CHD and prior diagnosis of HFrEF.

## Methods

### Study population

We reviewed the Mayo Adult Congenital Heart Disease Registry, and identified patients with CHD, biventricular physiology, and systemic LV. The Mayo Clinic Institutional Review Board approved this study. CHD diagnoses were classified as mild, moderate, or complex CHD based on contemporary guidelines.^18^ To account for differences in underlying CHD anatomy and physiology on HFrEF, the study cohort was classified into the following anatomic/physiologic subgroups: 1) right heart/right ventricular outflow tract lesions; 2) left heart lesions; 3) shunt lesions; and 4) others. Supplemental Table 1 shows underlying CHD diagnoses in the different anatomic/physiologic subgroups.

From this cohort, we identified consecutive patients who were diagnosed with HFrEF and had ≥1 year of follow-up between January 1, 2003, and December 31, 2023. HFrEF was defined as clinical features consistent with HF (symptoms [dyspnea, orthopnea, paroxysmal nocturnal dyspnea, fatigue, and pedal edema), signs [elevated jugular venous pressures, chest crackles, and pedal edema], and evidence of congestion such as elevated N terminal pro-B-type brain natriuretic peptide) in a patient with LVEF ≤40%.^2^^,^^19^ The first echocardiogram performed within the study showing LVEF ≤40% was considered the baseline echocardiogram, the subsequent echocardiogram performed 12 months (6-18) after the baseline echocardiogram was designated as the follow-up echocardiogram, and temporal change in LVEF was defined as the difference between the 2 echocardiograms. HFimpEF was defined as HF with baseline LVEF ≤40%, and a subsequent absolute increase ≥10%, leading to LVEF >40% at follow-up echocardiogram.^20^, ^21^, ^22^ The patients who did not meet the criteria for diagnosis of HFimpEF were classified as having persistent HFrEF.

### Outcomes

The primary outcome was all-cause mortality, and the secondary outcome was cardiovascular events defined as the composite endpoint of HF hospitalization, heart transplantation, or all-cause mortality. Outcomes were assessed as time-to-event endpoints and ascertained from the date of follow-up echocardiogram to the outcome of interest, last clinical follow-up, or December 31, 2023.

Exploratory outcome was HFrEF relapse defined as temporal decline in absolute LVEF >10% leading to LVEF ≤40% in patients with HFimpEF. HFrEF relapse was assessed as time-to-event endpoint and ascertained from the date of follow-up echocardiogram to the outcome of interest, last clinical follow-up, or December 31, 2023.

### Echocardiography

Comprehensive 2-dimensional, Doppler, and speckle tracking echocardiography was performed according to contemporary guidelines. Offline image analysis was performed in all patients by research sonographers using the standardized protocol for image analysis in the Mayo Adult Congenital Heart Disease Registry Imaging Core Laboratory.^23^

LV end-diastolic volume was derived from manually tracing the endocardial borders at end-diastole representing the maximal volume and LV end-systolic volume was derived from manually tracing the endocardial borders at end-systole representing the minimum volume. LVEF was assessed using the biplane Simpson method using the web-based Echocardiography Information Management System developed at Mayo Clinic to facilitate echocardiogram analysis, interpretation, and reporting.^24^ The LV volumes were indexed to body surface area. LV reverse remodeling was defined as temporal change (absolute Δ) in LV indices from baseline echocardiogram to the echocardiogram obtained at 1-year follow-up, whereby positive values signify a temporal increase, whereas negative values signify a temporal decrease.

### Data collection

Clinical data obtained within 6 months from baseline echocardiogram were used to define the baseline characteristics of the cohort. These include demographic indices, anatomic indices, comorbidities, laboratory indices, and hemodynamic indices (echocardiography, cardiac magnetic resonance imaging, and cardiac catheterization). We reviewed the HF medication list at baseline and follow-up encounters, to calculate the GDMT score for each patient at these encounters.^25^ ΔGDMT score was calculated as the difference between GDMT score at baseline and 1-year encounters (GDMT at the 1-year follow-up minus GDMT at baseline encounter) whereby positive values signify a temporal increase in GDMT use (GDMT uptitration), whereas negative values signify a temporal decrease in GDMT use. The HF medications and point system used for calculating GDMT score are shown in Supplemental Table 2.^25^ The GDMT score has not been validated in the CHD population.

### Statistical analysis

Data were presented as mean ± SD, median (Q1, Q3), and count (%). Between-group comparisons were based on Fisher exact test, unpaired t-test, or Wilcoxon rank sum test, as appropriate.

Multivariable logistic regression analysis was used to identify clinical indices associated with HFimpEF. The variables used in univariable models were selected based on clinical relevance and included demographic indices, CHD severity, CHD anatomic/physiologic groups, comorbidities, HF therapy (baseline GDMT score, ΔGDMT scores, and cardiac resynchronization therapy), and echocardiography-derived LV and right ventricular (RV) indices. Variables with *P* < 0.10 on univariable analyses were used to create the multivariable model, and the final variable selection was based on stepwise backwards selection, with *P* < 0.10 as the criterion for a variable to remain in the model. Subgroup analyses were performed to assess the relationship between GDMT use and HFimpEF using bivariate logistic regression analysis.

The cumulative incidences of all-cause mortality as well as cardiovascular event were estimated using Kaplan-Meier analysis, and between-group comparisons were based on log-rank test. Cox regression analysis was used to assess the relationship between HFimpEF, GDMT score, and outcomes (death and cardiovascular events). The proportionality of hazards was confirmed via graphical inspection of log-minus-log plots and Schoenfeld residuals. Covariate selection was based on the similar criteria as described previously. Exploratory analysis was performed to determine the incidence of HFrEF relapse among patients in the HFimpEF group, and the association between HFrEF relapse and cardiovascular events (HFrEF relapse was modeled as time-dependent covariate). All statistical analyses were performed with BlueSky Statistics software (version. 7.10; BlueSky Statistics LLC), and JMP statistical software (version 18.1.0; JMP Statistical Discovery LLC). *P* value <0.05 was considered to be statistically significant.

## Results

### Baseline characteristics

Of the 327 patients with prior diagnosis HFrEF, 63 (19%) had HFimpEF, whereas 264 (81%) had persistent HFrEF. Of the 327 patients, 76 (23%) had mild CHD, 215 (66%) had moderate CHD, and 36 (11%) had complex CHD. Supplemental Table 2 shows the underlying CHD diagnoses. There was no significant difference in CHD severity or CHD anatomic/physiologic group distribution between the 2 groups. Table 1 compares the baseline characteristics between the HFimpEF and persistent HFrEF groups. Although both groups had similar LVEF on baseline echocardiogram (LVEF 35% ± 4% vs 31% ± 6%; *P* = 0.16 for HFimpEF vs HFrEF, respectively), the HFimpEF group had better LV global longitudinal strain (−14% ± 3% vs −11% ± 3%, *P* = 0.04) compared to the HFrEF group, suggesting better LV systolic function. In addition, the HFimpEF group had greater use of HF medications and cardiac resynchronization therapy compared to the HFrEF group. Otherwise, there were no other significant between-group differences in clinical, echocardiographic, cardiac magnetic resonance imaging, and cardiac catheterization data (Table 1).

**Table 1:** Baseline Characteristics

	HFimpEF (n = 63, 19%)	HFrEF (n = 264, 81%)	*P* Value
Demographic indices			
Age (years)	45 ± 16	46 ± 16	0.54
Male	43 (68%)	160 (61%)	0.26
Body mass index (kg/m^2^)	26.4 ± 6.6	28.2 ± 7.5	0.06
CRT	12 (19%)	35 (13%)	<0.001
CHD severity			
Simple/mild	17 (27%)	59 (22%)	0.24
Moderate	36 (57%)	179 (68%)	
Complex/severe	10 (16%)	26 (10%)	
CHD anatomic/physiologic groups			
Right heart/RVOT lesion	29 (46%)	99 (38%)	0.46
Left heart lesion	11 (17%)	52 (20%)	0.78
Shunt lesion	18 (29%)	89 (34%)	0.43
Others	2 (3%)	3 (1.1%)	0.23
Comorbidities			
Hypertension	17 (27%)	101 (38%)	0.09
Coronary artery disease	37 (14%)	7 (11%)	0.53
Diabetes	5 (8%)	34 (13%)	0.26
Chronic kidney disease III-V	7 (11%)	37 (14%)	0.53
Atrial fibrillation	21 (33%)	76 (29%)	0.48
Laboratory indices			
GFR (ml/min/1.73 m^2^)	84 (67, 99)	79 (53, 96)	0.16
MELD-XI	11.3 (9.4, 13.9)	11.2 (9.4, 14.3)	0.86
NT-proBNP (pg/mL)	602 (219, 1984)	793 (293, 2,709)	0.42
GDMT			
Beta-blockers	38 (60%)	125 (47%)	0.06
ACEI/ARB	42 (66%)	107 (41%)	<0.001
ARNI	13 (21%)	29 (11%)	0.04
MRA	15 (24%)	36 (14%)	0.03
SGLT2i	11 (18%)	32 (12%)	0.25
Echocardiographic data			
Systemic indices			
LA reservoir strain (mL/m^2^)	21 ± 14	20 ± 11	0.13
LV end-diastolic volume (mL/m^2^)	82 ± 39	88 ± 35	0.38
LV end-systolic volume (mL/m^2^)	52 ± 31	56 ± 28	0.72
LV stroke volume index (mL/m^2^)	34 ± 15	33 ± 16	0.34
LV ejection fraction (%)	35 ± 4	31 ± 5	0.16
LV longitudinal strain (%)	−14 ± 3	−11 ± 3	0.04
≥ Moderate mitral regurgitation	8 (13%)	31 (12%)	0.83
Cardiac index (L/min/m^2^)	2.65 ± 1.031	2.69 ± 0.94	0.71
Nonsystemic indices			
RA reservoir strain (%)	19 ± 12	22 ± 12	0.41
RA mean pressure (mm Hg)	11 ± 6	10 ± 5	0.25
RV systolic pressure (mm Hg)	46 ± 20	48 ± 21	0.52
RV free wall strain (%)	−18 ± 8	−19 ± 6	0.42
≥ Moderate tricuspid regurgitation	21 (33%)	72 (27%)	0.34
Cardiac MRI	N = 22	N = 51	
LV end-diastolic volume (mL/m^2^)	80 ± 39	192 ± 42	0.44
LV end-systolic volume (mL/m^2^)	46 ± 27	68 ± 39	0.31
LV ejection fraction (%)	40 ± 15	41 ± 12	0.72
RV end-diastolic volume (mL/m^2^)	95 ± 35	113 ± 49	0.29
RV end-systolic volume (mL/m^2^)	57 ± 27	68 ± 39	0.31
RV ejection fraction (%)	40 ± 15	41 ± 12	0.72
Cardiac catheterization data	N = 19	N = 86	
RA mean pressure (mm Hg)	13 ± 7	14 ± 7	0.75
PA mean pressure (mm Hg)	28 ± 12	32 ± 14	0.18
PA wedge pressure (mm Hg)	17 ± 8	18 ± 10	0.61
Cardiac index (L/min/m^2^)	2.48 ± 1.53	2.45 ± 1.39	0.64
PVR index (WU × m^2^)	3.68 (1.90, 6.68)	4.66 (3.71, 9.6)	0.05

Values are mean ± SD, median (Q, Q3), and n (%). Between-group comparisons were based on Fisher exact test, independent t-test, or Wilcoxon rank sum test, as appropriate.

ACEI/ARB = angiotensin-converting enzyme inhibitor/angiotensin-II receptor blocker; ARNI = angiotensin receptor/neprilysin inhibitor; CHD = congenital heart disease; CRT = cardiac resynchronization therapy; GDMT = guideline-directed medical therapy; GFR = glomerular filtration rate; HFimpEF = heart failure with improved ejection fraction; HFrEF = heart failure with reduced ejection fraction; LA = left atrium; LV = left ventricle; MELD-XI = model for end-stage liver disease excluding international normalized ratio; MRA = mineralocorticoid receptor antagonist; MRI = magnetic resonance imaging; NT-proBNP = N-terminal pro–B-type brain natriuretic peptide; PA = Pulmonary artery; PVR = pulmonary vascular resistance; RA = right atrium; RV = right ventricle; RVOT = right ventricular outflow tract; SGLT2i = sodium-glucose cotransporter-II Inhibitors.

### GDMT score

Table 2 compares GDMT scores between the 2 groups. Compared to the persistent HFrEF group, the HFimpEF group had higher GDMT score at baseline encounter (3.24 ± 1.06 vs 2.19 ± 0.88, *P* = 0.02), and at 1-year follow-up encounter (4.21 ± 1.13 vs 2.88 ± 0.92, *P* < 0.001), as well as higher ΔGDMT score (0.92 ± 0.36 vs 0.71 ± 0.29, *P* = 0.01). These suggest greater use of GDMT at baseline encounter, as well as greater GDMT uptitration between the baseline and 1-year follow-up encounters.

**Table 2:** GDMT Use at Baseline and Follow-Up Encounters

	HFimpEF (n = 63, 19%)	HFrEF (n = 264, 81%)	*P* Value
GDMT score at baseline			
1	8 (13%)	88 (33%)	0.009
2	8 (13%)	90 (34%)	
3	23 (37%)	50 (19%)	
4	11 (18%)	24 (9%)	
5	8 (13%)	11 (4%)	
6	5 (8%)	1 (0.4%)	
GDMT score at 1-y follow-up			
1	0	0	<0.001
2	5 (8%)	103 (39%)	
3	16 (25%)	139 (53%)	
4	14 (22%)	25 (10%)	
5	17 (27%)	4 (2%)	
6	11 (18%)	3 (1%)	
Mean GDMT score			
GDMT score at baseline	3.24 ± 1.06	2.19 ± 0.88	0.02
GDMT score at 1-y follow-up	4.21 ± 1.13	2.88 ± 0.92	<0.001
ΔGDMT score	0.92 ± 0.36	0.71 ± 0.29	0.01

Values are mean ± SD and count (%).

Abbreviations as in Table 1.

### LV reverse remodeling

Table 3 shows LV indices derived from the echocardiograms at baseline and 1-year encounters. The HFimpEF group had robust LV reverse remodeling as evidenced by temporal decrease in LV volumes, as well as a temporal increase in LV stroke volumes (absolute Δ 10 ± 6 mL/m^2^), EF (absolute Δ 14% ± 4%), and global longitudinal strain (absolute Δ 3% ± 1%). In contrast, the persistent HFrEF group had less robust LV reverse remodeling (Table 3).

**Table 3:** LV Function Indices at Baseline and Follow-Up Encounters

	HFimpEF (n = 63, 19%)	HFrEF (n = 264, 81%)	*P* Value
LV indices at baseline echo			
LV end-diastolic volume (mL/m^2^)	82 ± 39	88 ± 35	0.38
LV end-systolic volume (mL/m^2^)	52 ± 31	56 ± 28	0.72
LV stroke volume index (mL/m^2^)	34 ± 15	33 ± 16	0.34
LV ejection fraction (%)	35 ± 4	31 ± 5	0.16
LV longitudinal strain (%)	−14 ± 3	−11 ± 3	0.04
≥Mod mitral regurgitation	8 (13%)	31 (12%)	0.83
LV indices at 1-y follow-up echo			
LV end-diastolic volume (mL/m^2^)	74 ± 21	81 ± 28	0.22
LV end-systolic volume (mL/m^2^)	38 ± 13	49 ± 21	<0.001
LV stroke volume index (mL/m^2^)	41 ± 12	35 ± 14	0.06
LV ejection fraction (%)	49 ± 6	38 ± 7	<0.001
LV longitudinal strain (%)	−17 ± 4	−12 ± 3	<0.001
≥Mod mitral regurgitation	6 (10%)	28 (11%)	0.81
LV reverse remodeling			
ΔLV end-diastolic volume (mL/m^2^)	−8 ± 4∗	−7 ± 5	0.26
ΔLV end-systolic volume (mL/m^2^)	−14 ± 8∗	−7 ± 4∗	<0.001
ΔLV stroke volume index (mL/m^2^)	10 ± 6∗	2 ± 4	<0.001
ΔLV ejection fraction (%)	14 ± 4∗	7 ± 4∗	<0.001
ΔLV longitudinal strain (%)	3 ± 1∗	1 ± 2	<0.001

Values are mean ± SD, median (Q, Q3), and n (%). Between-group comparisons were based on Fisher exact test and independent t-test. LV stroke volume assessment was based on Doppler echocardiography using the continuity equation. LV reverse remodeling represents temporal change in LV indices from baseline echocardiogram to 1-year follow-up echocardiogram. This was calculated as LV function indices obtained at 1 year follow-up minus indices obtained at baseline echocardiogram whereby positive values signify temporal increase, whereas negative values signify temporal decrease.

FU = follow up; other abbreviations as in Table 1.

∗ Statistically significant change from baseline values.

Table 4 shows the correlates of HFimpEF. Higher baseline GDMT score (adjusted OR: 1.38 per unit increase in baseline GDMT score; 95% CI: 1.02-1.98; *P* = 0.03) and greater GDMT uptitration (adjusted OR: 1.86 per unit increase in GDMT uptitration; 95% CI: 1.33-2.96; *P* = 0.009) were associated with greater odds of HFimpEF. Subgroup analysis showed similar associations between GDMT and HFimpEF across CHD anatomic/physiologic subgroups, albeit a greater effect size (greater odds of HFimpEF per unit increase in GDMT use) in patients with left heart lesions (Table 5).

**Table 4:** Logistic Regression Analysis Showing Correlates of Improved LV Ejection Fraction

	Univariable Analysis		Multivariable Analysis	
	OR (95% CI)	*P* Value	OR (95% CI)	*P* Value
Demographic/anatomic indices				
Age (per 5 years)	0.97 (0.89-1.06)	0.55		
Male	1.39 (0.78-2.55)	0.26		
CHD severity				
Mild/simple	Reference			
Mod/complex CHD (vs simple)	0.86 (0.49-1.76)	0.11		
CHD anatomic/physiologic group				
Left heart lesions	Reference			
Right heart/RVOT lesions	1.12 (0.89-2.72)	0.24		
Shunt lesions	0.98 (0.82-1.16)	0.39		
Others	1.04 (0.39-6.98)	0.71		
Comorbidities				
Hypertension	0.57 (0.29-1.09)	0.10		
Coronary artery disease	0.95 (0.34-2.33)	0.90		
Atrial fibrillation	0.73 (0.40-1.35)	0.31		
HF therapy				
Baseline GDMT score	1.77 (1.38-2.32)	<0.001	1.38 (1.02-1.98)	0.03
ΔGDMT score	2.44 (1.94-3.12)	<0.001	1.86 (1.33-2.96)	0.009
CRT at baseline	1.39 (1.12-2.17)	0.008		
Baseline echo indices				
LV end-diastolic volume (per 5 mL/m^2^)	0.98 (0.94-1.02)	0.31		
LV end-systolic volume (per 5 mL/m^2^)	0.99 (0.95-1.03)	0.48		
LV stroke volume index (per 5 mL/m^2^)	0.97 (0.92-1.02)	0.28		
LV ejection fraction (per 5%)	0.97 (0.93-1.01)	0.13		
LV longitudinal strain (%)	0.97 (0.95-0.99)	0.01		
≥ Moderate mitral regurgitation	0.82 (0.33-1.28)	0.39		
RV free wall strain (%)	0.94 (0.91-0.97)	0.008		
≥ Moderate tricuspid regurgitation	0.84 (0.61-1.05)	0.12		

Covariate with *P* < 0.10 on univariable analysis were used to create the multivariable model. Final covariate selection in the multivariable model was based on stepwise backwards selection with *P* < 0.10 as the criterion for a variable to remain in the model.

HF = heart failure; other abbreviations as in Table 1.

**Table 5:** Bivariate Logistic Regression Analysis Showing Correlates of Improved LV Ejection Fraction Stratified Based on Anatomic/Physiologic Subgroups

	Right Heart/RVOT Lesions		Left Heart Lesion		Shunt Lesions	
	OR (95% CI)	*P* Value	OR (95% CI)	*P* Value	OR (95% CI)	*P* Value
Baseline GDMT score	1.33 (1.02-1.91)	0.03	1.58 (1.17-3.32)	0.003	1.27 (1.05-2.44)	0.01
Δ GDMT score	1.64 (1.12-2.58)	0.008	2.02 (1.21-4.63)	<0.001	1.93 (1.10-2.82)	0.006

Abbreviations as in Table 1.

### Outcomes

**Figure 1:**
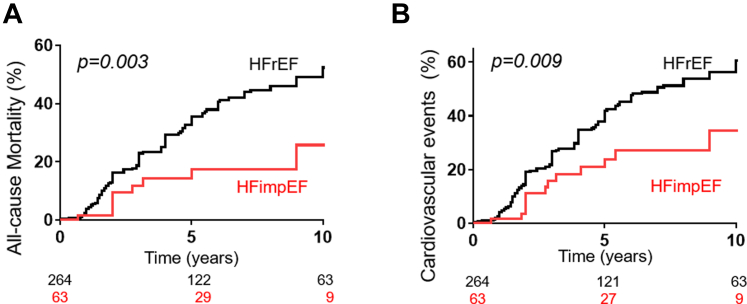
Kaplan-Meier Analysis of All-Cause Mortality and Cardiovascular Events Kaplan-Meier curves comparing cumulative incidence of (A) all-cause mortality and (B) cardiovascular events between patients with heart failure with improved ejection fraction (HFimpEF) (red) vs patients with persistent heart failure with reduced ejection fraction (HFrEF) (black).

Of the 327 patients, 124 (38%) were hospitalized for HF, 133 (41%) died, and 17 (5%) underwent heart transplantation during a median follow-up of 5.9 (3.1, 9.6), years. The 10-year cumulative incidence of all-cause mortality was lower in the HFimpEF group compared to the HFrEF group (27% vs 52%; *P* = 0.003) (Figure 1A). Compared to the persistent HFrEF group, being in the HFimpEF group was associated with a 28% reduction in all-cause mortality (adjusted HR: 0.72; 95% CI: 0.41-0.92; *P* = 0.009) after adjustment for demographic/anatomic indices, comorbidities, and end-organ dysfunction. Similarly, greater use of HF therapy as evidenced by higher GDMT score at 1-year follow-up was associated with a 17% reduction in all-cause mortality (adjusted HR: 0.83 per unit increase in GDMT score; 95% CI: 0.68-0.97; *P* = 0.005), after adjustment for demographic/anatomic indices, comorbidities, and end-organ dysfunction (Table 6). Notably, better RV systolic function on baseline echocardiogram was associated with lower risk of mortality as evidenced by 5% reduction in mortality per unit increase in RV global free wall strain (HR: 0.95; 95% CI: 0.91-0.99; *P* = 0.02).

**Table 6:** Cox Regression Analysis Showing Correlates of All-Cause Mortality

	Univariable		Multivariable	
	HR (95% CI)	*P* Value	HR (95% CI)	*P* Value
HF data				
HFimpEF (vs HFrEF)	0.69 (0.46-0.95)	0.005	0.72 (0.41-0.92)	0.009
GDMT score at 1-year follow-up	0.75 (0.64-0.87)	<0.001	0.83 (0.68-0.97)	0.005
Demographic/anatomic indices				
Age (per 5 years)	1.08 (1.04-1.14)	<0.001	1.07 (1.02-1.15)	0.03
Male	0.81 (0.56-1.41)	0.29		
Moderate/complex CHD	1.48 (1.03-2.41)	0.03		
CHD anatomic/physiologic group				
Left heart lesions	Reference			
Right heart/RVOT lesions	0.86 (0.72-1.06)	0.14		
Shunt lesions	1.02 (0.89-1.24)	0.54		
Others	1.06 (0.43-4.91)	0.66		
Echocardiographic indices				
Baseline LV longitudinal strain (%)	0.95 (0.91-0.99)	0.02		
Baseline RV free wall (%)	0.93 (0.90-0.96)	<0.001	0.95 (0.91-0.99)	0.02
Comorbidities				
Hypertension	1.36 (1.02-1.68)	0.01		
Coronary artery disease	1.56 (1.02-2.38)	0.04		
Diabetes	1.65 (1.06-2.63)	0.02		
Atrial fibrillation	1.34 (1.04-1.68)	0.009		
End-organ function				
GFR (per 10 mL/min/1.73 m^2^)	0.87 (0.82-0.93)	<0.001		
MELD-XI	1.06 (1.02-1.10)	0.004	1.05 (0.99-1.11)	0.09

Covariates with *P* < 0.10 on univariable analysis were used to create the multivariable model, and the final covariate selection was based on stepwise backwards selection with *P* < 0.10 required for a covariate to remain in the multivariable model.

Abbreviations as in Table 1.

Overall, 169 (52%) patients had at least 1 cardiovascular event. The 10-year cumulative incidence of cardiovascular events was lower in the HFimpEF group compared to the HFrEF group (34% vs 61%; *P* = 0.009) (Figure 1B). Compared to the persistent HFrEF group, being in the HFimpEF group was associated with a 28% reduction in cardiovascular events (adjusted HR: 0.74; 95% CI: 0.52-0.94; *P* = 0.003) after adjustment for demographic/anatomic indices, comorbidities, and end-organ dysfunction. Similarly, greater use HF therapy as evidenced by higher GDMT score at 1-year follow-up was associated with a 14% reduction in cardiovascular events (adjusted HR: 0.85 per unit increase in GDMT score; 95% CI: 0.73-0.97; *P* = 0.01), after adjustment for demographic/anatomic indices, comorbidities, and end-organ dysfunction (Table 7). Similarly, better RV systolic function on baseline echocardiogram was associated with a lower risk of cardiovascular event as evidence by 5% reduction in mortality per unit increase in RV global free wall strain (HR: 0.95; 95% CI: 0.92-0.98; *P* = 0.008).

**Table 7:** Cox Regression Model Showing Correlates of Cardiovascular Events

	Univariable		Multivariable	
	HR (95% CI)	*P* Value	HR (95% CI)	*P* Value
HF data				
HFimpEF (vs HFrEF)	0.64 (0.47-0.93)	<0.001	0.74 (0.52-0.94)	0.003
GDMT score at 1-year follow-up	0.83 (0.71-0.95)	<0.001	0.85 (0.73-0.97)	0.01
Demographic/anatomic indices				
Age (per 5 years)	1.06 (1.03-1.10)	<0.001	1.04 (0.98-1.10)	0.09
Male	0.84 (0.56-1.38)	0.24		
Moderate/complex CHD	1.33 (1.06-2.06)	0.04		
CHD anatomic/physiologic group				
Left heart lesions	Reference			
Right heart/RVOT lesions	1.22 (0.91-1.48)	0.21		
Shunt lesions	0.93 (0.69-1.15)	0.43		
Others	1.02 (0.26-3.87)	0.31		
Echocardiographic indices				
Baseline LV longitudinal strain (%)	0.95 (0.92-0.98)	<0.001		
Baseline RV free wall (%)	0.94 (0.92-0.96)	<0.001	0.95 (0.92-0.98)	0.008
Comorbidities				
Hypertension	1.34 (1.03-1.52)	0.009		
Coronary artery disease	1.41 (1.02-2.22)	0.03		
Diabetes	1.42 (0.99-2.73)	0.07		
Atrial fibrillation	1.42 (1.08-1.72)	<0.001		
End-organ function				
GFR (per 10 mL/min/1.73 m^2^)	0.91 (0.84-0.96)	<0.001		
MELD-XI	1.07 (1.03-1.11)	<0.001	1.05 (1.01-1.09)	0.01

Covariates with *P* < 0.10 on univariable analysis were used to create the multivariable model, and the final covariate selection was based on stepwise backwards selection with *P* < 0.10 required for a covariate to remain in the multivariable model.

Abbreviations as in Table 1.

### Exploratory analysis

Of the 63 patients in the HFimpEF group, 17 (27%) had HFrEF relapse during a median follow-up of 5.1 (2.8, 8.4), years, yielding a 5-year incidence of 26%. There was no significant between-group difference in age (43 ± 14 vs 47 ± 15 years, *P* = 0.33), baseline LVEF (37% vs 34% ± 4%, *P* = 0.16), GDMT score at baseline encounter (3.31 ± 0.94 vs 3.18 ± 0.91, *P* = 0.12), and GDMT at 1-year follow-up (4.46 ± 1.07 vs 4.13 ± 0.99, *P* = 0.08) between the patients with vs without HFrEF relapse, respectively. HFrEF relapse was associated with 23% increase in the risk of cardiovascular events (unadjusted HR: 1.23; 95% CI: 1.04-1.41; *P* = 0.02) compared to patients without HFrEF relapse.

## Discussion

In this study, we assessed the prevalence, correlates, and prognostic implications of HFimpEF in adults with CHD, biventricular physiology and systemic LV. We also assessed the incidence and prognostic implications of HFrEF relapse in patients with HFimpEF. The main findings are as follows: 1) of the 327 patients with initial diagnosis of HFrEF, 19% met the criteria for HFimpEF, and GDMT use was associated with greater odds of HFimpEF; 2) HFimpEF was associated with a 26% reduction in all-cause mortality, and 28% reduction in cardiovascular events on multivariable analysis; and 3) of the 63 patients with HFimpEF, 27% had HFrEF relapse, and HFrEF relapse was associated with 23% increase in cardiovascular events (Central Illustration).

**Central Illustration:**
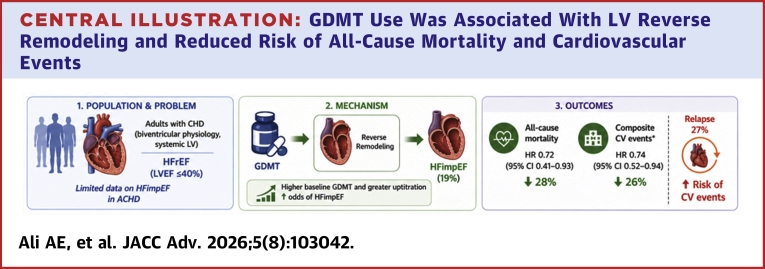
GDMT Use Was Associated With LV Reverse Remodeling and Reduced Risk of All-Cause Mortality and Cardiovascular Events ACHD = adult congenital heart disease; CHD = congenital heart disease; CV = cardiovascular; GDMT = guideline-directed medical therapy; HFimpEF = heart failure with improved ejection fraction; HFrEF = heart failure with reduced ejection fraction; LV = left ventricle.

Several studies have reported on the prevalence and outcomes of HFimpEF in patients with HFrEF due to acquired heart disease.^21^^,^^26^ In a meta-analysis of 9 studies including 9,491 patients with HFrEF, He et al^26^ observed that 23% of patients with HFrEF developed HFimpEF within 4 years of follow-up of 4 years, and the patients with HFimpEF had lower risk of mortality and HF hospitalization compared to patients with persistent HFrEF. Similarly, Min et al^21^ observed that 31% of patients with HFrEF developed HFimpEF with 12 months of HFrEF diagnosis, and that the patients with HFimpEF had a lower incidence of HF hospitalization (23% vs 45% per year) and mortality (6% vs 11% per year) compared to those with persistent HFrEF. However, 25% to 50% of patients with HFimpEF develop significant decline in LVEF (HFrEF relapse) within 5 years, and the patients with decline in LVEF have higher risk of mortality compared to those with decline in LVEF.^21^^,^^26^ Although the etiology of HFrEF relapse is multifactorial, Min et al^21^ observed that reduction or discontinuation of GDMT was associated with HFrEF relapsed and mortality. The results of these prior studies from the acquired heart disease population are consistent with the findings from our study.

In contrast to the robust data from the acquired heart disease population, the relationship between GDMT and improvement in LVEF and clinical outcomes in adults with CHD is less well defined.^11^, ^12^, ^13^^,^^15^, ^16^, ^17^ Nederend et al^15^ and Fusco et al^17^ reported improvement in systemic ventricular EF, 6-minute walk distance, and congestion following initiation of GDMT. Neijenhuis et al^11^ observed a 3-fold reduction in HF hospitalization without significant change in systemic ventricular EF following the initiation of sodium-glucose cotransporter-2 inhibitor in patients already on GDMT. However, Maurer et al^16^ did not observe any improvement in systemic ventricular EF and functional capacity following initiation of GDMT in CHD patients. These variations in outcome following initiation of GDMT in different studies are related to heterogeneity in the clinical characteristics of the CHD patients enrolled in these studies, and differences in the intensity of GDMT used in the different studies.^11^, ^12^, ^13^^,^^15^, ^16^, ^17^ The clinical response to GDMT will depend on systemic ventricular morphology and EF, as well as the intensity of HF therapy in the different studies.^11^, ^12^, ^13^^,^^15^, ^16^, ^17^ The current study overcame these limitations by restricting the inclusion criteria to CHD patients with morphologic LV and HFrEF, and by assessing intensity of HF therapy using the standardized GDMT score. Although the proportion of patients with HFimpEF was only 19% in the current study, this may be related to the strict criteria for improved LVEF in this study. Most studies defined improvement in LVEF as absolute increase in LVEF >10% or absolute increase in LVEF >5% leading to LVEF >40%.^20^, ^21^, ^22^ In this study, we used very strict criteria of absolute increase in LVEF greater than ≥10% leading to LVEF >40%.

The HF guidelines recommend cardiac resynchronization therapy in patients with HFrEF who remain symptomatic despite optimal medical therapy based on robust data from the acquired heart disease population demonstrating an association between cardiac resynchronization therapy, and improved outcomes.^1^ However, we did not observe an association between cardiac resynchronization therapy and HFimpEF in the current study. This is likely related to the study design whereby the baseline characteristics of the patients with vs without cardiac resynchronization therapy. Notably, in a prior study from our group using propensity matching to control for baseline characteristics, we demonstrated an association between cardiac resynchronization therapy, LV reverse remodeling, improvement in NT-proBNP, and improved survival.^27^

HF is the clinical manifestation of myocardial systolic and/or diastolic dysfunction, which in turn is driven by cellular and extracellular pathologic remodeling.^20^^,^^28^^,^^29^ Cellular remodeling is characterized by cardiomyocyte hypertrophy, metabolic dysregulation and disruption in protein expression and cellular signaling, whereas extracellular remodeling is characterized by changes in extracellular matrix such as increasing collagen deposition and fibrosis.^20^^,^^28^^,^^29^ Collectively, these microscopic changes lead to altered myocardial architecture and function, leading to systolic and/or diastolic myocardial dysfunction.^20^^,^^28^^,^^29^ GDMT use is associated with the restoration of cardiomyocyte size and reverse remodeling of extracellular architecture leading to improvement in LV chamber geometry and function (ie, improvement or normalization of LVEF).^20^^,^^28^^,^^29^ Despite overt improvement or normalization of LVEF, patients with HF still have dysregulation of cardiomyocyte function, progressive erosion of the native 3-dimensional organization of the extracellular matrix, and increased neurohormonal activation.^7^^,^^20^^,^^28^^,^^29^ Hence, HFimpEF represents a state of remission rather than recovery, with patients being susceptible to recurrent LV dysfunction in response to hemodynamic stressor or withdrawal of HF therapy.^20^^,^^28^^,^^29^ This may explain the observed HFrEF relapse in 27% of our cohort, which is consistent with a 25% to 50% prevalence of HFrEF relapse observed in the acquired heart disease population.^7^^,^^21^^,^^22^^,^^26^^,^^30^

### Study limitations

Study limitations include retrospective study design, which in turn, has inherent selection and ascertainment bias. Furthermore, we were unable to ascertain compliance with medical therapy due to the retrospective study design, and hence unable to determine etiology of HFrEF relapse.

## Conclusions

GDMT use was associated with improved LVEF (HFimpEF), and patients with HFimpEF had lower risk of mortality and cardiovascular events compared to patients with persistent HFrEF. Despite these improvements, patients with HFimpEF remain at risk for progression of LV systolic dysfunction (HFrEF relapse), which in turn is associated with higher risk of cardiovascular events. These findings support GDMT optimization in CHD patients with HFrEF, and ongoing clinical and imaging surveillance in this population.

Perspectives **COMPETENCY IN MEDICAL KNOWLEDGE:** GDMT use was associated with improved LVEF (HFimpEF), and patients with HFimpEF had lower risk of mortality and cardiovascular events compared to patients with persistent HFrEF. Despite these improvements, patients with HFimpEF remain at risk for progression of LV systolic dysfunction (HFrEF relapse), which in turn is associated with higher risk of cardiovascular events.

**TRANSLATIONAL OUTLOOK:** These findings support GDMT optimization in CHD patients with HFrEF, and ongoing clinical and imaging surveillance in this population.

## Funding support and author disclosures

Dr Egbe is supported by 10.13039/100000050National Heart, Lung, and Blood Institute (NHLBI) grants (R01 HL158517, R01 HL160761, and R01 HL162830). The MACHD Registry is supported by the Al-Bahar Research grant. All other authors have reported that they have no relationships relevant to the contents of this paper to disclose.
