# Supplementary material for: Heart Failure With Improved Ejection Fraction in ACHD With Biventricular Physiology and Systemic Left Ventricle
Source: JACC Adv. 2026 Jul 21;5(8):103042. doi: 10.1016/j.jacadv.2026.103042 (PMC13396863; doi:10.1016/j.jacadv.2026.103042)
Supplement: Supplemental Tables 1 and 2 [file mmc1.pdf]

Supplementary Table S1: CHD Diagnoses

CHD Diagnosis	All HFrEF (N=327, 4%)	HFimpEF (N=63, 19%)	Persistent HFrEF (N=264, 81%)	p
**Right heart/RVOT lesions**	**128 (39%)**	**29 (46%)**	**99 (38%)**	**0.46**
Tetralogy of Fallot	63 (19%)	11 (17%)	52 (20%)	
Ebstein	28 (9%)	5 (8%)	23 (8%)	
Pulm valve stenosis	15 (5%)	5 (8%)	10 (4%)	
Pulmonary atresia -IVS	4 (1.1%)	2 (3%)	2 (0.8%)	
Truncus arteriosus	3 (0.9%)	0	3 (1.1%)	
TGA- arterial switch op	6 (1.8%)	3 (5%)	3 (1.1%)	
TGA- Rastelli op	3 (0.9%)	0	3 (1.1%)	
Double outlet RV	6 1.8%)	3 (5%)	3 (1.1%)	
**Left heart lesions**	**87 (27%)**	**14 (22%)**	**63 (24%)**	**0.78**
Coarctation of aorta	44 (14%)	8 (13%)	36 (14%)	
Subaortic stenosis	4 (1.2%)	0	4 (1.5%)	
Aortic valve stenosis	37 (11%)	6 (10%)	31 (12%)	
Cor triatriatum	2 (0.6%)	0	2 (0.8%)	
**Shunt lesions**	107 (33%)	18 (29%)	89 (34%)	**0.43**
PAPVR	24 (11%)	2 (3%)	22 (8%)	
Atrial septal defect	33 (10%)	5 (8%)	28 (11%)	
Ventricular septal defect	37 (11%)	9 (3%)	28 (9%)	
Atrioventricular canal defect	13 (4%)	2 (3%)	11 (4%)	
Others	5 (1.5%)	2 (3%)	3 (1.1%)	**0.23**

Abbreviations: AS: Aortic stenosis; CHD: Congenital heart disease; HFrEF: Heart Failure with reduced ejection fraction; HfimpEF: Heart failure with improved ejection fraction; IVS: Intact ventricular septum; RV: Right ventricle; RVOT: Right ventricular outflow tract; TGA: Transposition of great arteries; PAPVR: Partial anomalous pulmonary venous return.

Supplementary Table S2: GDMT Score

Drug class	Dose	Points
**Beta blockers**		
	None	0
	<50% maximum dose	1
	≥50% maximum dose	2
**ACEI/ARB**		
	None	0
	<50% maximum dose	1
	≥50% maximum dose	2
**ARNI**		
	None	0
	Any dose	3
**MRA**		
	None	0
	Any dose	2
**SGLT2i**		
	None	0
	Any dose	1
**Maximum score**		8

Abbreviations: ACEI/ARB/ARNI: Angiotensin converting enzyme inhibitor / angiotensin 2 receptor blocker/ angiotensin receptor/neprilysin inhibitor; GDMT: Guideline directed medical therapy; MRA: Mineralocorticoid receptor antagonist; SGLT2i: Sodium-glucose cotransporter-2 inhibitor
